# 肿瘤标志物CYFRA21-1在非小细胞肺癌诊断中的价值

**DOI:** 10.3779/j.issn.1009-3419.2010.12.07

**Published:** 2010-12-20

**Authors:** 俊东 谷, 新卓 王, 辉 赵, 思伟 朱, 燕军 温, 洪瑞 许, 莉 李, 军 陈, 清华 周

**Affiliations:** 1 100121 天津，天津市人民医院胸外科 Department of Thoracic Surgery, Tianjin People's Hospital, Tianjin 300121, China; 2 100121 天津，天津市人民医院肿瘤科 Department of Oncology, Tianjin People's Hospital, Tianjin 300121, China; 3 300052 天津，天津医科大学总医院，天津市神经病学研究所 Department of Neuropatholosy, Neurology Institute, Tianjin Medical University General Hospital, Tianjin 300052, China; 4 天津市肺癌研究所，天津市肺癌转移与肿瘤微环境重点实验室 Tianjin Key Laboratory of Lung Cancer Metastasis and Tumor Microenviroment, Tianjin Lung Cancer Institute, Tianjin Medical University General Hospital, Tianjin 300052, China

**Keywords:** 肺肿瘤, 细胞角蛋白19片段抗原, ROC曲线, 诊断, Lung neoplasms, Antigen CYFRA21-1, ROC curve, Diagnosis

## Abstract

**背景与目的:**

细胞角蛋白19片段（cytokeratin 19 fragment, CYFRA21-1）是血液中的一种可溶性蛋白质，它可能是一种潜在的循环肿瘤标志物。本研究旨在探讨CYFRA21-1在非小细胞肺癌（non-small cell lung cancer, NSCLC）诊断中的应用价值。

**方法:**

采用酶联免疫吸附法分别检测107例NSCLC患者和51例肺部良性疾病患者外周血中CYFRA21-1含量，应用受试者工作曲线（receiver operaing characteristic curves, ROC）对检测结果进行临床评价。

**结果:**

NSCLC组织与肺良性疾病组外周血CYFRA21-1水平差别有统计学意义（*χ*^2^=47.343, *P* < 0.001）；以3.3 μg/L为诊断临界点，CYFRA21-1诊断NSCLC的敏感性和特异性分别为74.77%和76.47%，ROC曲线下面积为0.813 9；NSCLC各病理类型之间CYFRA21-1水平差别无有统计学意义（*χ*^2^=0.450, *P*=0.799）；外周血CYFRA21-1在广泛期（Ⅲb、Ⅳ）病例中明显高于局限期（Ⅰ、Ⅱ、Ⅲb）病例（*χ*^2^=7.057, *P*=0.008）。

**结论:**

CYFRA21-1作为肺癌肿瘤标志物具有相对较高的敏感性和特异性；血浆中CYFRA21-1水平升高提示肺癌患者可能已经发展为广泛期。

## Abstract (en)

**Background and objective:**

Cytokeratin-19 fragment (CYFRA21-1) is a soluble protein in serum, and may be a useful circulating tumor marker. The aim of this study is to investigate the diagnostic value of the peripheral blood CYFRA21-1 in non-small cell lung cancer (NSCLC).

**Methods:**

The levels of peripheral blood CYFRA21-1 were detected in 107 patients with NSCLC and 51 patients with benign pulmonary diseases by enzyme linked immunosorbent assay, and ROC curve was used to analyse the results.

**Results:**

Singificant difference of peripheral blood CYFRA21-1 levels was detected between the NSCLC group and benign pulmonary disease group (*χ*^2^=47.343, *P* < 0.001). At the threshold of 3.3 ng/mL, sensitivity and specificity of CYFRA21-1 as a serologic marker were 74.77% and 76.47%, respectively for any cancer. ROC curve showed that the under-curve area (AUC) of CYFRA21-1 was 0.813 9. There was no significant difference of CYFRA21-1 between subtypes of NSCLC (*χ*^2^=0.450, *P*=0.799). The peripheral blood CYFRA21-1 level was elevated significantly in the patients with extensive disease (Ⅲb, Ⅳ) compared with patients with limited disase (Ⅰ, Ⅱ, Ⅲb) (*χ*^2^=7.057, *P*=0.008).

**Conclusion:**

As a tumor marker CYFRA21-1 has relative high sensitivity and specificity for the diagnosis of NSCLC. Elevated peripheral blood CYFRA 21-1 levels were usually indicated extensive disease of NSCLC.

目前在多数发达国家，肺癌是最常见的恶性肿瘤，每年全球肺癌的新发病例超过120万，因此死亡的人数高达100万^[1]^。根据世界卫生组织（World Health Organization, WHO）定期公布的资料^[2]^显示，肺癌的发病率和死亡率在世界各国均呈明显上升趋势。尽管肺癌可以进行外科手术、放射和药物治疗，但其总的5年存活率仍在15%以下。肿瘤标志物包括癌胚抗原、神经烯醇化酶、鳞状细胞抗原等，在肿瘤患者外周血中的浓度明显升高，可用于肿瘤的早期诊断、预后判断及疗效监测。在肺腺癌中大多可以检测到有免疫活性的癌胚抗原，但其特异性不高^[3]^；神经烯醇化酶是神经元和神经内分泌细胞所特有的一种酸性蛋白酶，存在于人类所有细胞中，在大多数小细胞肺癌患者中其水平往往升高^[4]^，对肺癌患者连续监测血浆中神经烯醇化酶水平可以反映其体内肿瘤的发展变化情况^[5]^，但神经烯醇化酶同样存在特异性不高的问题，偶尔在非肿瘤性疾病者也有升高。鳞状细胞癌抗原是一种由子宫颈鳞状细胞组织萃取的一种醣蛋白，其敏感性为33%-61%^[6]^。细胞角蛋白19片段（cytokeratin 19 fragment, CYFRA21-1）为40 kDa上皮细胞骨架蛋白，已有研究^[7]^显示它对非小细胞肺癌（non-small cell lung cancer, NSCLC）的敏感性和特异性均较高。本文的目的在于进一步研究CYFRA21-1诊断NSCLC的敏感性和特异性，判断临床分期与外周血CYFRA21-1水平的关系。

## 1 材料与方法

### 1.1 患者资料

选取2005年1月-2009年6月在天津市人民医院胸外科、肿瘤科及呼吸科住院治疗并最终经病理或细胞学确诊的初治NSCLC患者107例作为病例组；同时期住院的非肿瘤患者51例为对照组。采用酶联免疫吸附法分别检测NSCLC患者和肺部良性疾病患者的外周血中CYFRA21-1水平。NSCLC组中男性62例，女性45例，中位年龄61岁，年龄范围33岁-84岁。其中鳞癌51例，腺癌48例，其它类型NSCLC 8例（表 1）。良性疾病组51例，其中男性32例，女性19例，中位年龄48岁。慢性阻塞性肺疾病（chronic obstructive pulmonary diseases, COPD）19例，肺炎18例，特发性肺纤维化14例。

**1:** 本研究纳入的非小细胞肺癌患者的一般情况 Characteristics of the patients with non-small cell lung cancer (NSCLC) included in this study

Characteristic	*n* (%)
Gender	62/45
Male	62 (57.9%)
Female	45 (42.1%)
Histologic type	
Squamous cell carcinoma	51 (47.7%)
Adenocarcinoma	48 (44.9%)
Others	8 (7.5%)
Stage	
Ⅰ	21 (19.6%)
Ⅱ	41 (38.3%)
Ⅲa	16(15.0%)
Ⅲb	9 (8.4%)
Ⅳ	20 (18.7%)

### 1.2 方法

患者于清晨空腹采静脉血3 mL-5 mL，静置20 min后离心（3 000/min, 10 min），分离外周血，-20 ℃保存待检。CYFRA21-1检测试剂由瑞士罗氏公司生产。

### 1.3 结果判定

以外周血中CYFRA21-1的含量为3.3 μg/L作为Cut-off值，计算其诊断肺癌的敏感性和特异性。

### 1.4 统计处理

采用SPSS 16.0统计软件，各组资料呈偏态分布，以中位数表示数据的集中趋势，组间比较采用*Kruskal-wallis*检验。以Cyrfa21-1检测结果作图绘制ROC曲线，计算曲线下面积和95%置信区间，判定以Cyrfa21-1作为肿瘤标志物诊断肺癌的效果。绘制箱式图，用以反映外周血中Cyrfa21-1水平与肺癌病理类型及临床分期间的关系。

## 2 结果

### 2.1 诊断肺癌的敏感性和特异性

NSCLC组与肺良性疾病组外周血CYFRA21-1水平差别有统计学意义（*χ*^2^=47.343, *P* < 0.001）。外周血中CYFRA21-1诊断肺癌的敏感性和特异性分别为74.77%和76.47%，CYFRA21-1的曲线下面积为0.813 9（图 1）。

**1:**
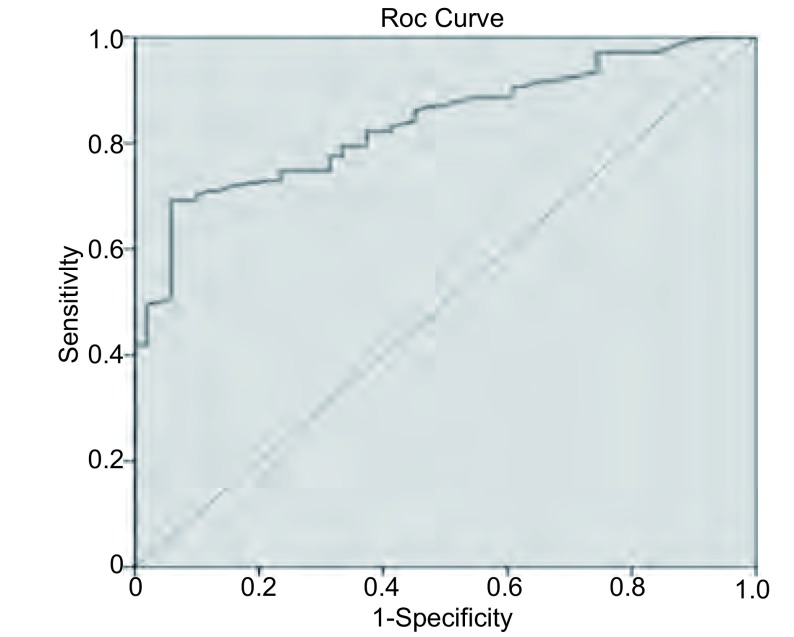
外周血肿瘤标志物CYFRA21-1的ROC曲线分析 Analysis of ROC curve of tumor marker CYFRA21-1 in peripheral blood. The areas under the ROC curve for CYFRA 21-1 was 0.813 9.

### 2.2 外周血中CYFRA21-1水平与NSCLC病理类型的关系

鳞癌、腺癌、其它病理类型和良性病变组患者外周血中CYFRA21-1的中位数分别为：6.40 μg/L（0.8 μg/L-32.1 μg/L）、6.10 μg/L（1.1 μg/L-22.1 μg/L）、5.7 μg/L（2.2 μg/L-16.98 μg/L）以及1.34 μg/L（0.1 μg/L-7.3 μg/L）（图 2）。NSCLC各病理类型之间CYFRA21-1水平差异无统计学意义（*χ*^2^=0.450, *P*=0.799）。

**2:**
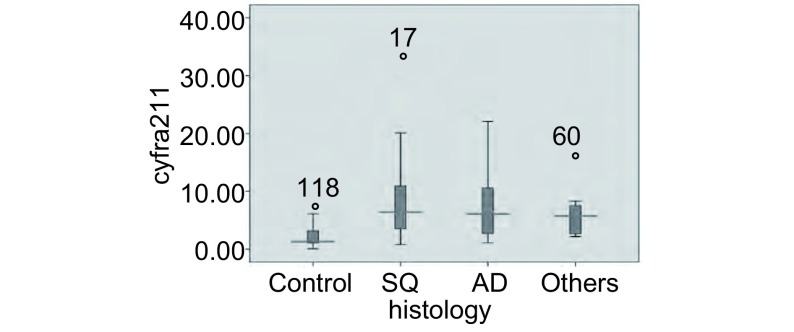
肺部良性疾病和NSCLC患者外周血CYFRA 21-1水平的箱式图 Distribution of CYFRA 21-1 according to the histology of NSCLC and benign pulmonary disease presented as multiple box plots showing median value, upper and lower quartile, minimum and maximum value.

### 2.3 外周血中CYFRA21-1水平与NSCLC临床分期的关系

Ⅰ、Ⅱ、Ⅲa、Ⅲb、Ⅳ期NSCLC患者外周血中CYFRA21-1水平的中位数（范围）分别为2.23 μg/L（0.86 μg/L-16.89 μg/L）、6.20 μg/L（0.80 μg/L-19.11 μg/L）、6.26 ng/mL（2.4 ng/mL-21.1 ng/mL）、10.11 μg/L（1.5 μg/L-20.1 μg/ L）和6.78 ng/mL（1.11 ng/mL-32.1 μg/L），随着病变的发展，外周血中CYFRA21-1平均浓度也在增高（图 3，图 4）。不同临床分期NSCLC患者外周血CYFRA21-1水平不相同（*χ*^2^=16.681, *P*=0.002）；其中局限期（Ⅰ、Ⅱ、Ⅲa）患者CYFRA21-1中位水平为5.60 μg/L，广泛期（Ⅲb、Ⅳ）患者CYFRA21-1中位水平为9.11 μg/L，两组比较差别有统计学意义（*χ*^2^=7.057, *P*=0.008）。

**3:**
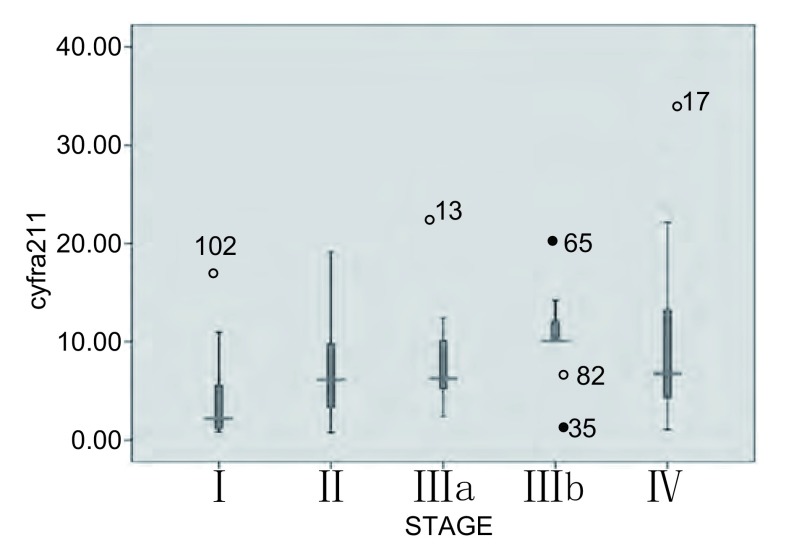
不同临床分期NSCLC患者外周血CYFRA 21-1水平的箱式图 Distribution of CYFRA 21-1 according to the clinical stage of NSCLC presented as multiple box plots showing median value, upper and lower quartile, minimum and maximum value.

**4:**
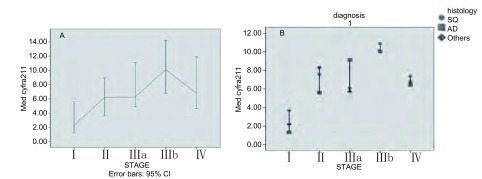
不同临床分期及病理类型NSCLC患者外周血CYFRA 21-1水平的线图。A：临床分期；B：病理类型。 Distribution of CYFRA 21-1 according to the clinic stage and histoloyg of NSCLC presented as line graph showing mean value and 95% confi- dence interval. A: clinic stye; B: histology.

## 3 讨论

当前在世界范围内，每年大约有超过120万人死于肺癌^[8]^，死亡人数超过了乳腺癌、前列腺癌和结肠癌的总和^[9]^。肺癌已成为一个重要的健康问题^[10]^。在过去的几十年里肺癌的死亡率并没有发生根本性的改变：20世纪50年代大约有5%的肺癌患者可以得到治愈，70年代增加至13%，但目前肺癌总的5年生存率也只有15%^[8]^。肺癌的不良预后可能与以下因素有关：首先，缺乏有效的早期诊断的方法；第二，大多数肺癌在得到确诊的同时往往已经发生转移；第三，术后经常复发；第四，对大多数化疗药物反应差。因此找到肺癌早期诊断的方法可能是提高肺癌5年生存率的关键^[11-13]^。外周血肿瘤标志物的异常往往早于影像学的异常，故对无临床相关症状患者，任何一项标志物的异常都可能具有提示意义。

CYFRA21-1作为上皮细胞角蛋白的成分之一，无明显的器官组织特异性，亦无肿瘤特异性。但是，上皮细胞癌变时，血中CYFRA21-1含量会升高^[14]^。细胞角蛋白在不同的上皮组织中表达水平是不同的，而且其表达不会因为上皮细胞的恶性转化而丢失，因此理论上可以通过检测细胞角蛋白的水平对肺癌的细胞类型加以区分^[15]^。本研究的结果显示肺癌组外周血CYFRA21-1水平明显高于肺部良性病变组（*χ*^2^=47.343, *P* < 0.001），但结果未发现外周血CYFRA21-1水平升高与组织病理学之间的关系（*χ*^2^=0.450, *P*=0.799）。

ROC曲线是肿瘤标志物临床应用中一种全面、准确评价诊断试剂的非常有效的方法。根据Swets^[16]^报道，面积在0.5以下时无诊断价值；面积在0.5-0.7时有较低的准确性；面积在0.7-0.9时有较高的准确性；面积在0.9以上时准确性最高。本结果显示CYFRA21-1的曲线下面积为0.813 9，提示CYFRA21-1可作为区别肺癌患者与正常人较好的指标。

从临床分期来看，NSCLC患者外周血中CYFRA21-1水平与病理分期存在一定的关系，随着病变的发展，外周血中CYFRA21-1平均浓度也在增高，但病变发展到Ⅳ期时外周血中CYFRA21-1水平不再增加反而有下降趋势，此结果与以往的研究^[17]^有所不同，分析原因可能是由于Ⅲb期患者数量较少（9例）所致，或是由其它未知原因所造成，需加大样本量进行进一步的研究。局限期（Ⅰ、Ⅱ、Ⅲa）病变患者外周血CYFRA21-1水平较低（中位数为5.60 μg/L），广泛期（Ⅲb、Ⅳ）病变患者外周血中CYFRA21-1水平普遍较高（中位数为9.11 μg/L），两组比较差别有统计学意义（*χ*^2^=7.057, *P*=0.008），提示外周血中CYFRA21-1水平较高的NSCLC患者可能已发展为广泛期病变，可能已失去手术时机。因此，对外周血中CYFRA21-1水平较高的NSCLC患者实施手术时应慎重，需对患者进行更为全面的术前检查，除外远处转移等广泛期病变的存在。
